# Occlusal plane canting: a treatment alternative using skeletal anchorage

**DOI:** 10.1590/2177-6709.24.1.088-105.sar

**Published:** 2019

**Authors:** Marcel Marchiori Farret

**Affiliations:** 1 Fundação para Reabilitação das Deformidades Crânio-Faciais - FUNDEF, Curso de Especialização em Ortodontia (Lajeado/RS, Brazil).; 2 Centro de Estudos Odontológicos Meridional - CEOM, Curso de Especialização em Ortodontia (Passo Fundo/RS, Brazil).; 3 Private practice (Santa Maria/RS, Brazil).

**Keywords:** Occlusal plane canting, Skeletal anchorage, Adult treatment

## Abstract

A canted occlusal plane is the cause of unaesthetic smile, and also represents a challenge, due to the complex orthodontic procedures involved in its treatment. The skeletal anchorage allows successful treatment of this asymmetry in the majority of cases, with less dependency on the patient cooperation and reducing the necessity of orthognatic surgery. Given this condition, this article aims at discussing the main aspects related to the diagnosis of occlusal plane canting, treatment plan, and orthodontic mechanics using skeletal anchorage either by mini-implants or miniplates. In this context, five cases will be reported, showing the main details related to the orthodontic mechanics used to correct the occlusal plane, avoiding side effects and successfully reaching treatment objectives and long-term stability.

## RESUMO

O plano oclusal inclinado é causa de um sorriso esteticamente desagradável e representa um desafio, devido à complexidade dos procedimentos ortodônticos envolvidos no seu tratamento. A ancoragem esquelética permite a correção da inclinação do plano oclusal, na maior parte dos casos, sem a necessidade de cirurgia ortognática e com menor dependência da colaboração dos pacientes. Com base nisso, o objetivo desse artigo é discutir os principais aspectos relacionados ao diagnóstico, plano de tratamento e mecânica ortodôntica envolvida no tratamento do plano oclusal inclinado com mini-implantes ou miniplacas. Nesse contexto, cinco casos serão apresentados, demonstrando os principais detalhes relacionados à mecânica utilizada na correção do plano oclusal inclinado com a utilização de ancoragem esquelética.

## INTRODUCTION

Asymmetric cases may be considered a huge challenge for orthodontists due to the complex mechanics and the uncertain stability of the treatment. Occlusal plane canting is one of the asymmetries that usually cause additional complexity in the treatment.^1^ ^-^ ^6^

During the time preceding the advent of skeletal anchorage, occlusal plane canting was normally treated with complex mechanics using elastics, asymmetric bends in the archwires, bite blocks, high-pull headgears, or orthognathic surgery, in cases with severe deviations.^2^ ^,^ ^3^ ^,^ ^6^ ^,^ ^7^ Recently, mini-implants and miniplates have been used as good options to correct occlusal plane deviations either in the frontal or the lateral view.^2,4,7-14^ Mini-implants are a good option in mild to moderate deviations, whereas miniplates are considered a good option when larger deviations are diagnosed or when a group of teeth must be moved in different directions at the same time.^2^ ^,^ ^8^ ^,^ ^9^ ^,^ ^15^

When the skeletal anchorage is indicated, a diagnosis is essential to determine where to insert the temporary anchorage device and which part should be intruded or extruded to eliminate canting.^2^ ^,^ ^12^ Furthermore, a number of details must be addressed in the mechanics to control the side effects, avoiding unexpected results at the end of the treatment.^3^ ^,^ ^9^ ^,^ ^14^

Based on the aesthetic relevance and frequency of the canting of the occlusal plane in orthodontic patients, this manuscript aims to discuss the considerable resources that may be used to establish the correct diagnosis and prepare the ideal treatment plan for cases of occlusal plane canting.

## DIAGNOSIS

In cases of canted occlusal plane, is essential to define which side should be intruded or extruded to level the plane. Usually the upper arch serves as the reference to the diagnosis through the exposure of the crowns and the gingiva, and the orthodontist must know all the aesthetic commandments to interpret the smile. Numerous resources may be used to evaluate how canting is established, as described below.^5^ ^,^ ^16^ ^-^ ^24^

### Photographs of smile

Photographs are the most traditional resources to study smile aesthetics, which are important keys to diagnose asymmetries in the occlusal plane. First, a photograph of a spontaneous smile must be captured to show the maximum elevation of the upper lip.^16^ ^,^ ^18^ ^,^ ^21^ ^,^ ^25^ ^,^ ^26^ The photograph of the smile during occlusion is a part of the regular orthodontic documentation and may be used to identify any deviation in the upper arch.^17^ ^,^ ^27^ ^,^ ^28^ Furthermore, another photograph may be captured, with a spontaneous smile and the mouth slightly opened, to evaluate the lower arch and the parallelism of the curvature of the upper arch with the lower lip. Capturing a series of smile photographs is recommended to better represent the dynamics of the smile, facilitating the identification of deviations from normality. Another important resource is the use of oral retractors for taking facial photographs in occlusion and with the mouth slightly open, also helping to identify deviations of the upper and lower occlusal planes in comparison to the face.

### Software

**Figure 1:**
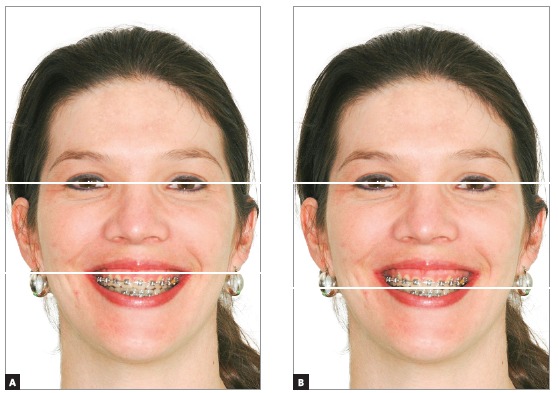
Bipupilar line transferred to the commissures region (A) and to the cuspid tip of tooth #23 (B), to verify the occlusal plane canting in both the posterior and anterior regions.

**Figure 2:**
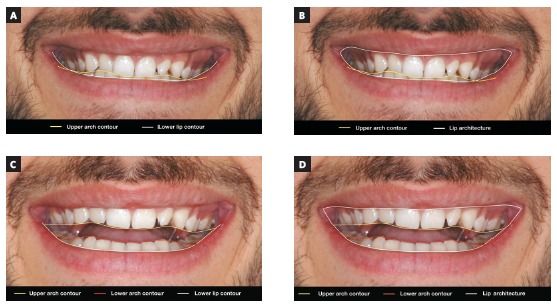
Possibilities of drawing reference lines, using software such as Keynote or PowerPoint, to verify the symmetry of the upper occlusal plane in relation to the lower lip (A) and in relation to the lip architecture (B) in occlusion. Reference lines to verify the symmetry of the upper and lower occlusal planes in relation to the lower lip (C) and in relation to the lip architecture (D) with open mouth.

Currently, with the aid of computers and software, such as PowerPoint or Keynote, the analysis of smiles can be facilitated by the use of reference lines.^5^ ^,^ ^18^ ^,^ ^21^ ^,^ ^27^ ^,^ ^29^ One of these lines is the bipupilar line, which may be transferred from the original position to the commissures region, to the gingival contour or tip of the cusp of one canine, or even the incisal edge of one incisor, depending on the necessity, being this method appropriated to evaluate either the anterior or posterior region of the upper arch. Other lines may be drawn following the contour of the upper arch, lower arch, lower lip or the labial architecture, to compare the symmetry among them. It is important to emphasize that some patients who show a cant of the occlusal plane also have an asymmetry in the labial architecture when smiling and this asymmetry should not be taken into account in the definition of the diagnosis and treatment plan, being therefore, the bipupilar line a more reliable reference in these situations (Figs 1 and 2). 

### Devices

Several devices may be used to facilitate the analysis and evaluation of asymmetries on the posterior region. Either a wooden tongue depressor or a metallic ruler can be used in the posterior region, with the patient in occlusion, allowing the analysis of the asymmetries in this region with greater precision.^2^ ^,^ ^17^ ^,^ ^27^

### Radiographies or tomographies

Other resources are the PA teleradiography and the tomography of the face, which must be used especially when severe skeletal asymmetries are present, and the treatment plan requires an orthognathic surgery as the main step to correct the canted occlusal plane.^3^ ^,^ ^12^ ^,^ ^16^ ^,^ ^30^ ^,^ ^31^

### Midline

**Figure 3:**
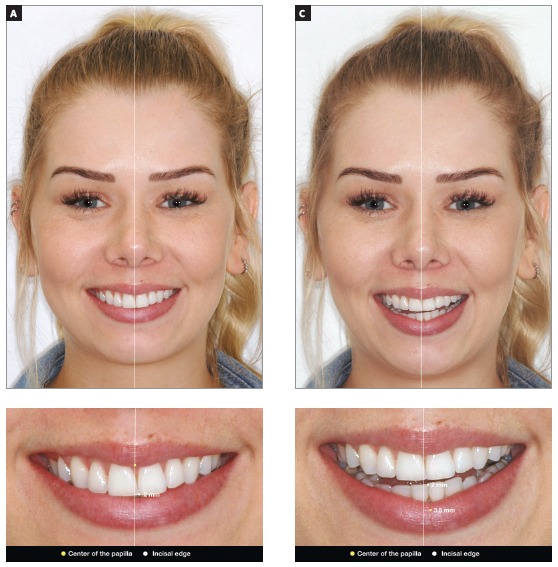
Definition of the midline in a patient with incorrect axial inclination of the anterior teeth. A) Facial photography of the smile in occlusion, and the facial midline traced; B) close-up view of the midline in the region of the papilla and incisal region - observe how there is a large discrepancy between the papilla and the incisal region, in comparison to the facial midline. C) Smile photograph with open mouth, and facial midline drawn; D) close-up view with midline in the papilla region and incisal region of the lower arch. It is important to highlight that, in situations of surgical correction of mandibular asymmetry, the definition of the lower arch midline should follow a different protocol, accompanying the center of the chin.

The diagnosis of the upper and lower midlines position in cases of occlusal plane asymmetries must follow different rules, in comparison with symmetric cases.^1^ ^,^ ^28^ ^,^ ^32^ Usually there is incorrect axial inclination of the anterior teeth associated with the cant of the occlusal plane, therefore, in these cases the professional must measure the dental midline in the papillar region, either in the upper or lower arch. Such requirement must be met because with the incorrect angulation of the anterior teeth, caused by occlusal plane canting, the midline measured in the incisal border does not represent the correct center of that group of anterior teeth. After correcting the occlusal plane, the teeth are uprighted according to the papilla, and not according to the incisal area. The scheme of Figure 3 shows how the upper and lower midlines should be diagnosed in patients with asymmetries.

## TREATMENT PLAN

Based on the diagnosis, the treatment plan for occlusal plane canting may follow different patterns, which are discussed below. 

### Unilateral intrusion of the upper arch

In the upper arch, when the diagnosis reveals good teeth and gingival display on one side and an excessive gingival display on the other side, this side must be intruded, followed by the extrusion of the antagonist teeth of the lower arch. After intrusion on the upper arch, the arch must be held in position, whereas the lower arch is extruded with intermaxillary vertical elastics, which are connected directly to the upper arch or occasionally connected to the skeletal anchorage device.

### Intrusion of the lower arch

In cases where the upper arch displays 100% of the crown without gingiva exposure in one side, whereas the other side the exposure of the crown is less than 100%, we should avoid intrusion on the upper arch, because the intrusion would reduce exposure on the good side, considerably impairing the aesthetics of the smile. In this case, the correction of the occlusal plane must begin with an intrusion on the lower arch, on the same side with reduced exposure of the crowns on the upper arch. After intrusion on the lower arch, this arch is stabilized, and the patient is advised to use vertical elastics on this side, to provoke the extrusion of the upper arch. 

### Combination of intrusion on both arches

When one side on the upper arch displays no gingiva and less than 100% of the crowns, whereas the other side shows the entire crowns and excess of gingiva, the option should be the combination of intrusion on the side with excess, and extrusion of the side where the crowns are incompletely exposed. However, previously, an intrusion on the lower arch on this side is necessary. Therefore, the correction of canting in such cases must start with the intrusion on both arches. When the extent of the intrusion is the same on the upper and lower arches, an extrusion on the opposite arch is unnecessary, because intrusion on both sides will correct the canting itself. When the extent of intrusion differs between the sides, vertical elastics might be occasionally necessary on one side to establish a good intercuspation. This option is preferably indicated in hyperdivergent patients, because only intrusive forces and little or no extrusive mechanics will be used.

## MECHANICS

### Mini-implants or miniplates?

Intrusion may be carried out with mini-implants when occlusal plane canting is slight or moderate, and no anteroposterior mechanics are necessary, for example, Class II correction. Miniplates are indicated when the canting is severe, because this device may receive a higher load and shows no risk for root contacting with screws during the intrusion - which can occur when using mini-implants. Miniplates are also a good option when, in addition to occlusal plane canting, a sagital correction must be performed in the same side. In these cases, movements in more than one direction can be performed, reducing the total time of treatment.

### Controlling the side effects

Although the mechanics is performed with the aid of skeletal anchorage, the side effects are not totally avoided and must be controlled by the orthodontist. The most common side effect, when the mechanics is performed only on the buccal side, is the buccal flaring of the intruded teeth, and it occurs due to the distance from the point where the force is applied and the center of resistance of the group of teeth, creating a moment of force and moving the crowns buccally. A tendency for crossbite is commonly observed on the opposite side, due to the rotation of occlusal plane, moving the crowns lingually on this side. One alternative to avoid these undesirable effects in the posterior region of the upper arch is to use a removable transpalatal bar (TPB), which avoids the overexpansion of the arch on the side of intrusion and aids in controlling the torque on both sides. Another option is to use buccal and palatal temporary anchorage device, thereby eliminating the need for TPB to control side effects on that side. Furthermore, during the intrusion, the use of 0.019 × 0.025-in stainless steel archwire is essential, allowing the control of the torque, with an accentuated buccal root torque on the side of the intrusion, whether a lingual root torque must be inserted on the opposite side, avoiding the tendency for crossbite. If the intrusion is performed on the lower arch, a lingual arch is the option to avoid the same side effects, associated with the same rectangular archwire and torque control as in the upper arch.

### Intermaxillary vertical elastics

Simultaneously performing intrusion and extrusion is not recommended, due to the impossibility of establishing the same force for both movements. After intrusion on the upper or lower arch, the extrusion on the opposite arch must be performed. First, the intruded region must be stabilized with metal ligature connected with a mini-implant or a miniplate and connected to the teeth or directly to the archwire. Afterward, the most common way for extrusion is the use of vertical elastics connecting the intruded teeth to the opposite arch or connecting the skeletal anchorage device to the opposite side. Similar in intrusion, the side effects are constantly present in the extrusion with elastics, showing a tendency for lingual crown movement, which should be avoided with the same 0.019 × 0.025-in rectangular stainless steel archwire. A lingual root torque must lie on the side of the extrusion, avoiding the lingual inclination of the crowns and buccal root torque on the opposite side, and avoiding the crossbite tendency on the other.

### Midlines

The upper and lower midlines usually are deviate from the facial midline in cases with occlusal plane canting. As discussed in the Diagnosis section, the correct midline must be defined through clinical examination and mainly by smile photographs. Mechanically, the midline measured in the incisal area of the incisors presents more significant changes during the treatment, whereas the midline measured in the papillar area shows a lower tendency to change. If an intrusion is performed on one side of the upper arch, the upper midline measured in the incisal region will shift considerably to the same side, whereas the midline in the papillar region will present less shifting. During extrusion, the midline in the incisal and papillar regions shift to the opposite side. Therefore, these changes must be carefully considered before starting the treatment, to avoid unexpected effects during treatment. If the upper and lower midlines are canted before treatment, coincidentally, the correction of occlusal plane canting will generate uprighted upper and lower midlines; however, those midlines will be deviated between them, and additional mechanics will be necessary to correct this deviation, increasing the total treatment time and probably upsetting the patient. Considering this condition before the beginning of the treatment, the orthodontist may plan additional anchorage devices or different mechanics to correct all deviations, reducing the total treatment time. The scheme of Figure 6 shows how the upper midline responds to the intrusion and extrusion movements used to correct the occlusal plane canting.

**Figure 4:**
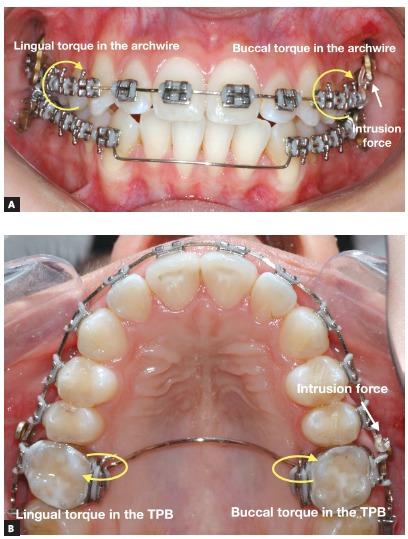
Scheme of control of the side effects during the mechanics to correct a canted occlusal plane with skeletal anchorage: A) Torque control in the archwire during the intrusion on the left side of the upper arch, B) auxiliary torque control through the transpalatal bar (TPB).

**Figure 5:**
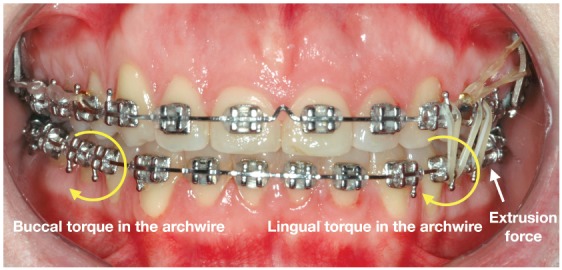
Torque control in the lower arch during the extrusion mechanics with intermaxillary elastics.

**Figure 6:**
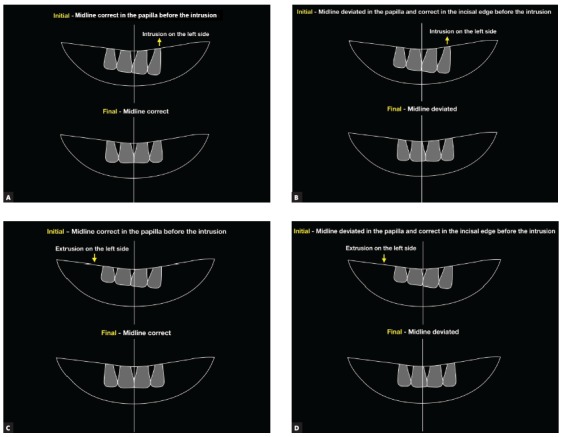
Effect on the upper midline provoked by the correction of occlusal plane: A) Correct midline in the region of the papilla at the beginning, and correct at the end of occlusal plane canting correction with intrusion; B) correct midline at the incisal edge at the beginning and deviated after occlusal plane canting correction with intrusion; C) midline correct in the papilla region at the beginning and correct at the end of the occlusal plane canting correction with extrusion; D) correct midline at the incisal edge at the beginning and deviated after the occlusal plane canting correction with extrusion.

## CASE 1

### Diagnosis and treatment plan

**Figure 7:**
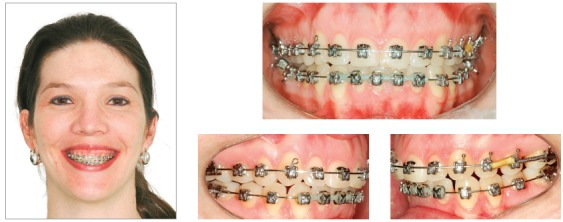
Initial photographs.

This case presents the sequence of an unsuccessful orthodontic treatment conducted for 3 years. A 29-year-old woman has been treated to correct a Class II, subdivision left malocclusion, with accentuated midline deviation, using unilateral intermaxillary elastics. The side effects of the long period using the elastics were occlusal plane canting, mainly in the region of canine and adjacent teeth, which can be identified in the frontal smile photograph. The upper midline was deviated 4 mm to the right, with slight inclination of the anterior teeth to the right, while the lower midline was correct. Similarly, the patient presented an accentuated gummy smile, with 6-mm of gingival display on the upper central incisors region. Intraoral analysis showed a Class II malocclusion on the left side, accentuated deviation between the midlines, and a 4-mm overjet, whereas the lower anterior teeth were rotated. The chief complaint of the patient was the gummy smile and occlusal plane canting, which according to her was non-existent before the first treatment (Fig 7).

Based on the diagnosis, the planned treatment was the use of two miniplates in the upper arch to correct occlusal plane canting, associated to Class II relationship correction. As the occlusal plane canting was caused by the first orthodontic treatment during the use of unilateral Class II elastics, it was decided to use a similar sagittal force; however, with upward direction, provoking an intrusion on the left side of the upper arch. As the Class II was more pronounced on the left side, the force should be higher in this side, allowing the correction of the midline as well. With the miniplates, it was possible to considerably reduce the total treatment time, because sagittal and vertical problems would be corrected at the same time and a group of teeth could be moved together. After orthodontic treatment, the patient would be refereed to periodontal surgery and rehabilitation of the anterior teeth.

### Treatment progress

**Figure 8:**
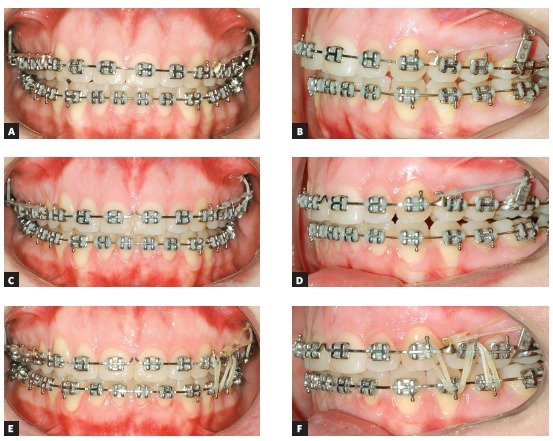
Photographs of treatment progress: A, B) beginning of the mechanics with miniplates with 400g of force in the left side, with an upward direction; and 150g in the right side, without vertical component; C, D) two months of mechanics and application of 200g of intrusive force in tooth #26 region; E, F) end of Class II and occlusal plane canting correction on the left side, stabilization with two elastics with intrusive and retraction force, and beginning of the use of intermaxillary elastics for extrusion of the lower arch.

**Figure 9:**
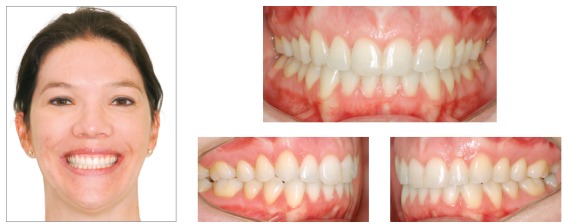
Final photographs after periodontal surgery and restorations.

All the brackets from the previous treatment were removed, and the orthodontic appliance was rebonded. Standard Edgewise brackets with 0.022 × 0.028-in slots were used, and alignment and leveling started with rounded nickel-titanium archwires (0.012 and 0.014-in), followed by 0.016-in to 0.019 × 0.025-in stainless steel archwires on both arches. One miniplate was installed on each side of the upper arch in the zygomatic pillar area, and the mechanics began with a retraction force (400 g/f) delivered by the elastic chains with upward direction on the left side and without vertical force on the right side (150 g/f). The force on the left side was higher, in order to correct the Class II in this side and correct the upper midline deviation. Two months later, a TPB was positioned, and a vertical force (200 g/f) was applied directly to tooth #26 to level the posterior region of the arch. In the fourth month of mechanics, the force was applied only on the left side and, after the correction of Class II relationship on the left side, vertical intermaxillary elastics were used only on the left side, to extrude the lower teeth (Fig 8). Occlusal plane canting and the gummy smile progressively reduced, as shown in the smile photographs. After achieving the treatment objectives, a period of 4 months has been considered before the debonding. After the orthodontic treatment, a periodontal surgery was performed. Then, new restorations were made on the upper incisors and the final results can be observed in the Figure 9.

## CASE 2

### Diagnosis and treatment plan

**Figure 10:**
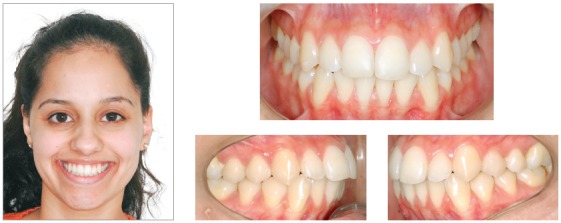
Initial photographs.

The patient was a 24-year-old woman who previously underwent orthodontic treatment for 3 years. The patient was unsatisfied with the incorrect angulation and excessive proclination of the anterior teeth, with crowding on the lower arch. Facial analysis revealed a convex profile and mandibular asymmetry, with deviation to the right side, which probably provoked an asymmetry on the upper arch. The analysis of the midline showed a 3-mm deviation in comparison with the facial midline, with angulation of the anterior teeth to the right side. Analysis of the intraoral photographs showed a Class I relationship on both sides (Fig 10). On this basis, one of the treatment options for this case was four premolar extractions. This option would encompass almost all the necessities of the case. However, the occlusal plane canting would remain uncorrected. Furthermore, the patient refused the extractions due to the spaces that would be created before closing with orthodontic mechanics. Thus, the best option considered was the use of skeletal anchorage to level the occlusal plane and obtain spaces to reduce the projection of the incisors and eliminate crowding in the lower arch. 

### Treatment progress

**Figure 11:**
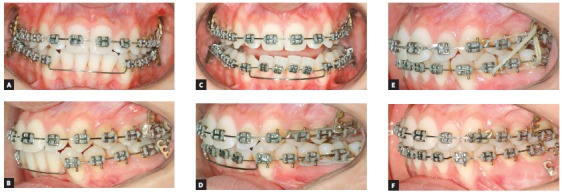
Treatment progress: A, B) Beginning of the mechanics with upper left side intrusion, C, D) end of upper intrusion and inclusion of lower incisors, after lower teeth retraction; E) beginning of mechanics with elastics connected directly to the miniplate and to the lower arch and F) end of the lower extrusion.

**Figure 12:**
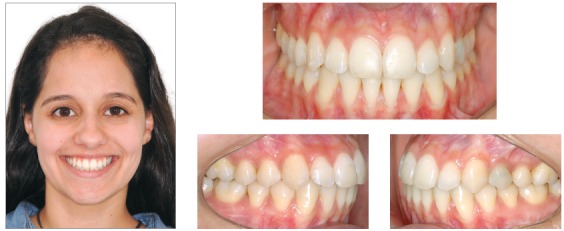
Final photographs.

Standard Edgewise brackets with 0.022 × 0.028-in slots were bonded on the upper and lower arches, except for the lower incisors. Alignment and leveling were performed from the 0.012 and 0.014-in nickel-titanium archwires through the 0.016, 0.018, and 0.020-in stainless steel archwires up to rectangular 0.019 × 0.025-in archwires. At this moment, four miniplates were positioned, one in each posterior region of the quadrants, and the intrusion on the left side of the upper arch started with elastomeric chains connected directly from the miniplate to the arch, with 200 g/f. After one month, the retraction of both arches with elastics connected from the miniplates to hooks welded over the arches was started. Three months after initiating the retraction, the lower incisors were bonded, and an overlaid 0.012-in nickel-titanium archwire was inserted to align and level the teeth. The treatment progressed until correction of the upper occlusal plane, and at this moment, this arch was stabilized with metal ligatures connected to the miniplate. The patient was instructed to use intermaxillary elastics directly connected to the miniplate in the upper arch and to the lower teeth, to promote the lower dentition extrusion on the left side (Fig 11). After the extrusion of the lower arch, the treatment was stabilized for 4 months before debonding. The final results of the treatment are shown in Figure 12. 

## CASE 3

### Diagnosis and treatment plan

**Figure 13:**
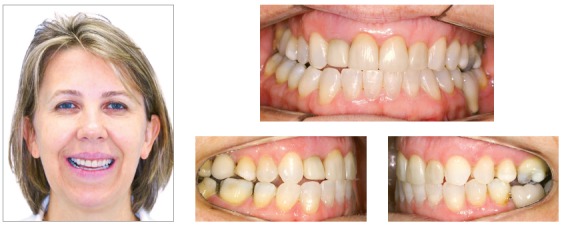
Initial photographs.

A 48-year-old woman sought for orthodontic retreatment, complaining about unaesthetic smile. The facial analysis revealed an increased lower third height, and the patient presented no passive lip seal. The profile was convex, and the smile aesthetics was impaired due to occlusal plane canting associated to incorrect angulation of the anterior teeth to the right side. Intraoral photographs showed: Class III relationship and crossbite on the right side; Class II relationship on the left side; upper midline deviated 1.5 mm to the right, and edge-to-edge relationship among incisors. The patient received implant-prosthetic rehabilitation on the right maxillary first molar, right maxillary lateral incisor, and left mandibular first molar (Figs 12 and 13). The first alternative considered was the surgical correction of the occlusal plane canting through the maxillary impaction on the left side and mandibular surgical rotation, which could lead to a counterclockwise rotation of the mandible, reducing the facial height and profile convexity. However, the patient refused the orthognathic surgery. Thus, this option was discarded. The second option considered was the combination of intrusion on the maxillary left side and mandibular right side with the aid of miniplates. This alternative could provoke the mandibular counterclockwise rotation and reduce the facial vertical pattern. Moreover, the Class III relationship would be corrected by means of distalization on the right side, eliminating the anterior edge-to-edge relationship, whereas the lower left second molar would be uprighted and supported by the implant on the first molar. The patient chose this treatment option.

### Treatment progress

**Figure 14:**
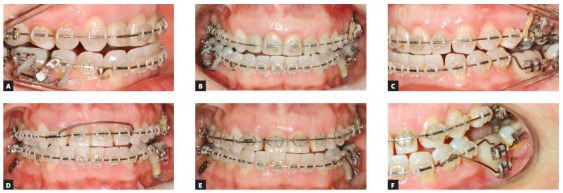
Photographs of treatment progress: A-C) Mechanics with miniplates for upper left intrusion and intrusion in the lower right side, associated with retraction, D) end of the intrusion with miniplates, E) intrusion of the left side of anterior region with cantilever connected to the implant of tooth #12 and F) extrusion of the left side of the lower arch, with aid of cantilever connected to the implant of the tooth #36.

**Figure 15:**
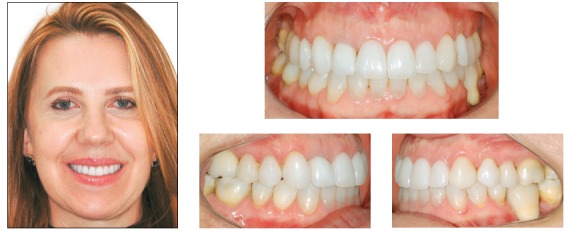
Final photographs.

The treatment started with alignment and leveling with 0.012 and 0.014-in nickel-titanium archwires, followed by 0.016 to 0.019 × 0.025-in stainless steel archwires with 0.022 × 0.028-in ceramic standard Edgewise brackets. After six months, a TPB was installed on the upper arch, and the patient was referred to a maxillofacial surgeon to insert a miniplate on the zygomatic area on the left side of the maxilla and on the external oblique ridge on the right side of the mandible. One month after the surgery, power arms were adapted on the tubes fitted to the miniplates, and elastomeric chains were connected from the power arms to the archwire. Only an intrusion force was present on the upper arch, whereas on the lower arch, intrusion and retraction forces were delivered by the elastics, to correct the Class III relationship and anterior crossbite. On the lower arch, the lingual arch was discarded due to the implant on the left side; the implant was used with a contracted archwire during the intrusion, avoiding the overexpansion of the arch on the right side. After 4 months of intrusion, the posterior occlusal plane canting was almost totally corrected. However, the upper arch presented occlusal plane canting that was localized mainly on the left side of the anterior region. Therefore, a cantilever made with 0.017 × 0.025-in titanium-molybdenum wire was inserted in the bracket of the upper right lateral incisor, which was an implant, and connected to the region between the upper left lateral incisor and canine, with an intrusion force of 100 g/f. Then, the lower arch would be extruded on the left side. The option was bonding another bracket over the buccal surface of the mandibular left first molar, which was also an implant, and connecting a similar cantilever to the maxillary arch, delivering an extrusion force of 100 g/f. Another step was performed on the posterior region of the arch, close to the implant, provoking an extrusion of the premolar region (Fig 14). Four months after the orthodontic treatment, the patient was referred to perform a gingival surgery from the maxillary right second premolar to the maxillary left premolar, increasing the crown lengthening and establishing symmetry among the gingival contour of the maxillary teeth, and after the surgery, the patient started her aesthetic rehabilitation. The final results can be observed in Figure 15. 

## CASE 4

### Diagnosis and treatment plan

**Figure 16:**
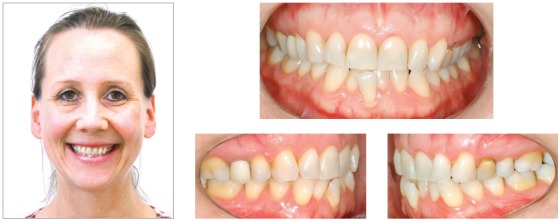
Initial photographs.

A 32-year-old woman sought for orthodontic treatment, complaining about the crowding on the anterior region of the mandibular arch. The smile analysis revealed an occlusal plane canting, with more gingiva displayed on the left side. Intraoral analysis showed: Class I molar relationship, slight Class III canine relationship, correct upper midline (measured in the papilla), and lower midline deviated 2 mm to the right. The lower arch discrepancy was -7 mm, and the upper arch discrepancy totaled -2 mm. Analysis of Bolton discrepancy revealed a 2-mm excess on the anterior region of the lower arch (Fig 16). 

The treatment plan involved intrusion of the upper left side with mini-implant positioned between the premolars, as the initial periapical radiography showed a good space on that region. Given the increased lower discrepancy and the Bolton discrepancy with lower excess, the decision was to extract tooth #41 and close the space with tooth #31 in the midline. 

### Treatment progress

**Figure 17:**
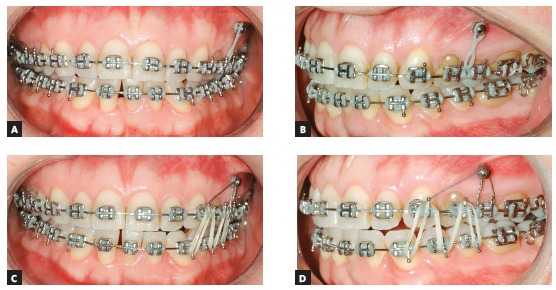
Photographs of treatment progress: A, B) beginning of upper arch intrusion and C, D) stabilization of the upper arch in the mini-implant and extrusion of the lower arch with intermaxillary elastics.

**Figure 18:**
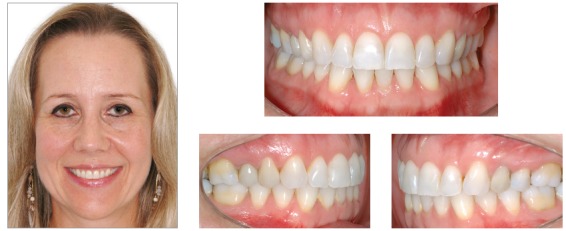
Final photographs.

Treatment started with bonding of standard Edgewise brackets on both arches and extraction of tooth #41. Alignment and leveling were performed with rounded nickel-titanium archwires until the 0.019 × 0.025-in stainless steel archwires. At this moment, a mini-implant was inserted between teeth #24 and #25, and a small force (50 g/f) was immediately applied from the mini-implant to the upper arch with an elastic chain. One month after, the force was increased to 200 g/f, and the intrusion was monitored monthly. After three months, canting of the upper arch was corrected; this arch was stabilized with metallic ligatures, as shown in Figure 17. To promote the lower teeth extrusion, intermaxillary elastics (3/8-in) were connected from the upper to the lower teeth, in the shape of an "M'. After correcting the lower arch cant, the treatment was stabilized for four more months before the removal. After the orthodontic treatment, the patient underwent oral rehabilitation with the new prosthesis and restorations (Fig 18).

## CASE 5

### Diagnosis and treatment plan

**Figure 19:**
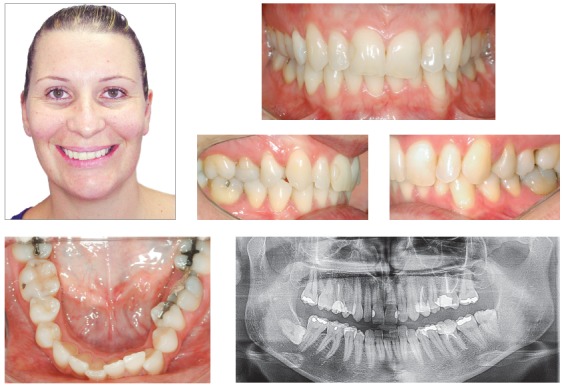
Initial photographs.

Female patient, 36 years of age at the beginning of treatment, whose facial analysis revealed asymmetry, with mandibular deviation to the left side. Due to mandibular asymmetry, there was also an asymmetry of lips in both resting and smiling. In addition, in the smile it was possible to identify an asymmetry of the occlusal plane, with good exposure of the upper teeth on the right side and reduced exposure of the upper teeth on the left side. The upper midline was coincident in the papilla and deviated 1.5 mm to the left in the incisal region, and the lower midline was coincident in the incisal region and 2 mm deviated to the left in the papilla region. In the intraoral analysis, it was observed a Class II relationship on the right side and Class I on the left side, crowding in the anterior region of both arches and a subgingival fracture of the tooth #36. On the panoramic radiograph, it was possible to verify that tooth #36 presented extensive restoration and poorly conducted endodontics, which would require further intervention. In addition, the patient had the tooth #38 in the mouth and with good crown and root shape, and the tooth #48 was impacted and in a position of difficult traction (Fig 19).

The planned treatment was the extraction of teeth #36 and #45, to create spaces in the lower arch to align the incisors, and the rest of the space would be used for loss of anchorage with the help of Class II elastics. After the closure of the lower spaces, a mini-implant would be used in the left side of the lower arch, to provoke the intrusion of this region, followed by the extrusion of the upper teeth with the aid of intermaxillary elastics.

### Treatment progress

**Figure 20:**
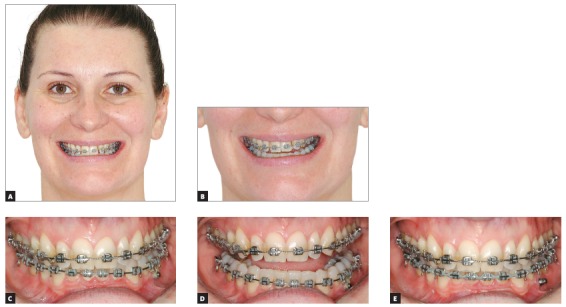
Photographs of treatment progress: A, B) analysis of the smile before the beginning of the occlusal plane canting correction; C, D) end of the lower occlusal plane canting correction, with the aid of mini-implant; and E) end of the extrusion of the upper arch with intermaxillary elastics.

**Figure 21:**
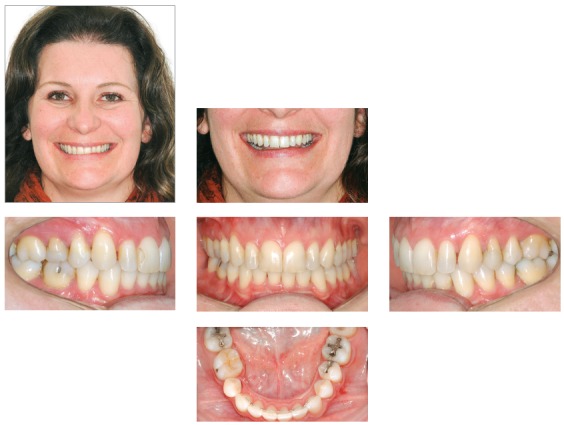
Final photographs prior to rehabilitation.

Standard Edgewise brackets with 0.022 × 0.028-in slots were initially bonded in both arches, except for the lower incisors. Alignment and leveling was conducted with round nickel-titanium (0.012 and 0.014-in) arches, followed by 0.016 to 0.019 × 0.025-in steel wires in both arches. At that moment, the teeth #36 and #45 were extracted and the closure of the spaces started with elastic chains connecting an anterior segment on each side in tie-together and the teeth to be mesialized (#37 and #46). Thus, it would be obtained distalization of the anterior segment to obtain spaces for the incisors, and mesialization of the posterior teeth would also be obtained to close the spaces. After three months of mechanics with elastics, the lower incisors were bonded and aligned and leveled with superimposed nickel-titanium archwires. The closure of the residual spaces of the lower arch was then performed with rectangular arches and T-loops, and with the aid of Class II elastics. After the spaces were closed, a mini-implant was inserted between the teeth #34 and #35, and the process of intrusion was started with the attached elastic chain from the mini-implant to the lower arch, which had buccal root torque in the left side and lingual torque on the right side. After the intrusion, the lower arch was stabilized with metal ligatures and elastic chain, and the patient proceeded to use 3/8-in intermaxillary elastic in a shape of "M", to provoke upper left extrusion (Fig 20). After the upper extrusion, some rebondings and bends were made, defining the spaces to fill the upper lateral incisors, then the appliance was removed. After removal, the patient was referred to perform bleaching procedures and restorations on anterior teeth. Figure 21 shows the final orthodontic result, before the bleaching and restorations on the anterior teeth.

## FINAL CONSIDERATIONS

Vertical asymmetries have always represented a greater complexity in the orthodontic treatment of adult patients. Usually in cases of slight deviations, with the use of conventional mechanics, these asymmetries were treated in a limited way or with important side effects in the final result. In cases of moderate to severe deviations, the association with orthognathic surgery was essential to obtain satisfactory results. The possibility of using skeletal anchorage in these cases allowed the correction of important asymmetries without side effects and reducing the necessity for orthognathic surgery. However, it is fundamental to perform a correct diagnosis in these cases, in order to plan the positioning of the skeletal anchorage device and consequently of the region to be moved, correcting the asymmetry. In addition, the orthodontist must have control of unwanted effects from the mechanics, avoiding the prolongation of the orthodontic treatment or even unsatisfactory results.
